# Studies on healthy and neoplastic tissues using positron annihilation lifetime spectroscopy and focused histopathological imaging

**DOI:** 10.1038/s41598-020-68727-3

**Published:** 2020-07-17

**Authors:** B. Zgardzińska, G. Chołubek, B. Jarosz, K. Wysogląd, M. Gorgol, M. Goździuk, M. Chołubek, B. Jasińska

**Affiliations:** 1 Institute of Physics, Maria Curie-Sklodowska University, Pl. Marii Curie-Skłodowskiej 1, 20-031 Lublin, Poland; 2 Diagnostic Techniques Unit, Faculty of Health Sciences, Medical University of Lublin, Al. Racławickie 1, 20-059 Lublin, Poland; 3 Department of Neurosurgery and Pediatric Neurosurgery, Medical University of Lublin, Al. Racławickie 1, 20-954 Lublin, Poland; 4 Medical Faculty, Medical University of Lublin, Al. Racławickie 1, 20-059 Lublin, Poland

**Keywords:** Imaging, Cancer imaging, Biophysics, Medical research, Oncology, Materials science, Biological physics, Techniques and instrumentation, Condensed-matter physics, Structure of solids and liquids, Nuclear physics

## Abstract

Samples of healthy and neoplastic myometrial tissues were investigated using Positron Annihilation Lifetime Spectroscopy (PALS). Meaningful differences between normal and diseased tissues were observed for each patient. The differences were also clearly visible for various kinds of lesions in each patient. The set of lifetimes and intensities obtained from PALS was correlated with the histopathological examinations of the same fragments of tissues. Strong coincidence between PALS parameters and histopathological findings was observed only in the case of a very precise correlation of the investigated area in both techniques. Measurements and discussion presented here were carried out to develop a method for measuring the sub-nanometric structure of human tissues. This kind of investigation, using positron probe, creates an opportunity of a new application in Positron Emission Tomography (PET).

## Introduction

Positron (e+, the electron antiparticle) and positronium (Ps—a hydrogen-like atom of bounded e− and e+) have been used to study the structure of matter over last few decades. The processes of formation and annihilation of e+ and Ps in gases, liquids and solids have been quite well understood and described by a mathematical apparatus, contributed to a better understanding of the physico-chemical properties and structure of matter at the nanometer scale. From the studies of a various materials (organic, inorganic, porous, etc.), it is known that positronium atoms locate in defects and empty spaces of the medium (so-called free volumes, FV, spaces with reduced electron density)^1–3^. The size of such a FV and its chemical environment are correlated with the average ortho-Positronium (o-Ps, the triplet state of Ps with parallel spins of e− and e+) lifetime (τ_o-Ps_). This is the basis of positron porosimetry allowing to determine the void sizes in the range 0.2 ÷ 100 nm^4–10^. The generally understood chemistry of the medium and the concentration of FVs are reflected in the intensity of annihilating components, especially the value of o-Ps intensity (I_o-Ps_).

Positrons are used in medical diagnostics, in a popular medical imaging technique PET (Positron Emission Tomography). On the other hand, positronium giving the possibility to determine the lesion location and giving additional data about processes occurring in human tissue is not applied in PET technique yet. Our knowledge about the processes involving Ps in complex systems, like biological ones, is still very limited. Only a few papers devoted to the antimatter probe in such systems were published, and up to now there are no papers related to a systematic study of human tissue (due to the problems with an access to the test material and the degree of diversity and complexity of the tissue). Positron annihilation lifetime spectroscopy (PALS) in application to the living biological systems was discussed in a very limited number of papers, focusing mainly on readily available samples. Among others, the animal and human tissues and cells were investigated: rat muscles and brain^11^, mouse skin^12^, human tissues (skin and hair)^13–16^, human cell culture^17^. The analysis of the impact of humidity was carried out on simple organisms—yeast^18^. In literature^19^ the theoretical considerations and modeling can be found. The results of above-cited papers are charged by various factors affecting the response of the PALS technique and do not allow to draw general conclusions. The first systematic research of human tissues by PALS technique was presented just recently by Lublin and Cracow groups^20–22^.

The possibility of implementation the positronium properties into PET technique, extending its abilities in diagnosis was reported recently^23^. Nowadays we are on stage of developing a new tomograph—J-PET (Jagellonian PET) which, among a number of its functionalities, contains also the possibility to measure the Ps lifetime spectra^23–28^. This means that we get the opportunity to study the structure, which depends on the type of examined tissue as well as any possible lesions occurring in it. In a broader perspective, this will give us the opportunity to develop a new, non-invasive imaging technique, supplementary to those based on the standardized uptake value (SUV index), commonly used in PET findings’ analysis. Moreover, it should be noted that the interpretation of the test results will provide a new opportunity to understand the tissue alteration, should the mechanisms of formation and interaction of e+ and Ps in the structures of the human body be comprehensively studied.

In the case of complex systems, the nano-structure is no longer a simple issue and thus requires the adoption of a series of initial assumptions and simplifications. In general, it is necessary to reference and correlate with wider knowledge and results obtained by other techniques, while taking into account both micro and macro scales. Complex systems—like human tissues, although being made of cells of similar structure, origin, metabolism and functionality, show a large morphological and physiological diversity. The mechanisms leading to disordering in life functions and the growth of cells of such tissues are intensively studied, because they can affect the progress of understanding the diseases (including cancer) and the formation of body dysfunctions^29,30^. It is known that a change of the functionality of cells is accompanied by a chemical change in their environment and a change in their structure. Both factors will affect the probability of e+ and Ps formation and annihilation, which supports the use of e+ as an excellent probe carrying information reflecting processes occurring in tissue at the cognitively unique nanometer scale.

According to the literature, nearly 80% of women can be affected by uterine fibroids^31^. The exact number of cases depends on the target population, the patient’s age and diagnostic techniques used. They are the most common benign tumors of the reproductive organs occurring in women of reproductive age. The asymptomatic fibroids are visualized during ultrasound examinations of up to 10% of women in the first trimester of pregnancy^32^. According to reports^33–35^, the incidence of fibroids among Caucasian women in the American population was 35% at the age of 35 and over 70% at the age of 50. Fibroids cause significant morbidity and deterioration in quality of life^36^. Some uterine fibroids remain asymptomatic, but most of them cause symptoms such as abnormal bleeding, pain, feeling of pressure or urinary complications^33^. Due to the high rate of occurrence, severity of symptoms and frequent need for treatment, the removal of fibroids (among others, by hysterectomy) is the most commonly performed gynecological surgery. Therefore, the availability of tissue material that can be obtained for scientific research is high. An important advantage of this choice is also the availability of the reference sample—as a result of the hysterectomy, the entire organ is removed, and therefore both the changed (leiomyomas) and healthy tissues are available. Pilot studies made using the PALS technique^20,21^ have already shown that the interpretation of o-Ps parameters will be possible if the result obtained for diseased tissue can be related to the results obtained for healthy tissue. Therefore, the samples are taken in pairs: abnormal and normal (neoplastic and healthy). At this point, it is worth to note that the problem of access to reference samples will not occur in the case of PET. From the point of view of the future application in PET, the possibility to distinguish between the normal tissue and the kind of lesion is very important.

Clinical diagnosis in conjunction with ultrasound imaging and visual assessment of uterine disease during hysterectomy and sampling is, however, insufficient to unambiguously assess the nature and extent of lesions of samples subjected to PALS analysis. In order to fill the scale gap between the assessment of macroscopically diagnosed lesions and those obtained on the scale of nanometers, it was decided to relate the obtained results to histopathological examinations completing the scale of microscopic evaluation of tissue changes. Histopathological examination ultimately determines the type of pathological changes in tissues taken from the patients. In the case of malignant tumors, the result of this study determines the further treatment of these patients^37^. However, the histopathological examination result depends on the quality and representativeness of the tissue material. The biggest problem exists when the tumor is inoperable and deeply located, which makes it difficult to take a sample for histopathological examination. At each stage of tissue processing an error may occur, which will impede or prevent the correct interpretation of the microscopic image.

## Experimental

### Clinical diagnosis and sampling

**Figure 1:**
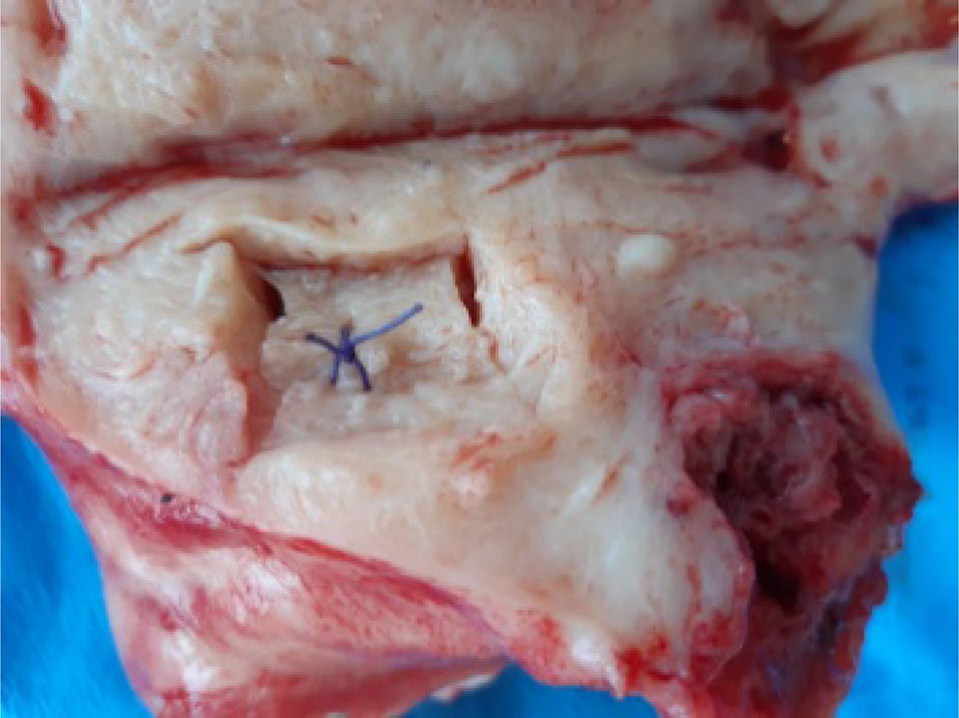
The place of sampling in the organ immediately after removal from the body to allow proper histopathological evaluation of tissues adjacent to the examined site.

Two patients were qualified for surgery in accordance with proper diagnostic and therapeutic procedures. These included: 1. anamnesis, 2. clinical examination, 3. supplementary examinations, including imaging (in the case of first patient vaginal ultrasound, in the second case vaginal ultrasound and abdominal computed tomography), 4. qualification for the surgical procedure, 5. patients' informed consent for the procedure and inclusion in PALS research. The sites of tissue collection for the research, both in healthy and diseased areas, were selected macroscopically by an experienced gynecologist, who did not participate directly in the surgery. This occurred in the operating room immediately after removal of the organ from the patient's body (Fig. 1). Two slices of each tissue samples, separated by a polycarbonate plate were placed directly into steal measuring chamber (Fig. 3a), properly coded and placed in a thermoinsulated container at room temperature. The material was delivered to the research center within 30 min. The first patient, 51 years old, was diagnosed with numerous uterine myomas and left ovarian cyst, with recurrent excessive menstrual bleeding and a secondary iron deficiency anemia. Surgery was the patient’s treatment of choice. Previously, a histopathological biopsy was performed on the uterine cavity, excluding endometrial cancer. The second patient, 66 years old, was initially diagnosed with a disseminated neoplastic process with probable genesis in an ovarian neoplasm, ascites and peritoneal metastases within greater momentum. Preliminary diagnosis was confirmed by histopathological examination performed after surgery.

### Positron annihilation lifetime spectroscopy (PALS)

Positron annihilation lifetime spectroscopy (PALS) measurements were conducted using the digital coincidence spectrometer with BaF_2_ scintillation detectors. The time resolution curve was approximated by single gaussian with a full width at half maximum (FWHM) of about 190 ps. Positron emitting ^22^Na radioactive source, with the activity of 0.38 MBq, was enclosed in 8 µm-thick Kapton envelope. The ^22^Na source was placed in an additional Teflon frame, which allows its repeated safe operation (Fig. 3b). Thus prepared source with frame can be safely placed in the polycarbonate plate hole (visible in Fig. 3a) and then inserting the source in the central part of the chamber with sample by moving the plate immediately before the measurement. Such *source on moving plate* solution was used, among others for safety reasons (doctors placing samples in the chamber and persons transporting samples to the PALS laboratory do not have contact with radioactive sources, on the other hand the chamber remains closed from the moment when the sample is placed until the end of the PALS test, so the laboratory's contact with the sample is kept to a minimum) and for maintaining the measurement standard (the measurement geometry is maintained, placing the source near the sample just before starting the PALS measurement eliminates the occurrence of potential patterns of earlier irradiation of the sample). The samples were kept in a constant temperature of 21 °C, during both, transport from the hospital to PALS laboratory and the measurement. The PAL spectra were measured each time for 1.5 h, which ensured that sample aging does not affect the results. The total count number obtained for each sample was 1.5 ÷ 2 million. The spectra were analysed using LT 9.2 program^38^, assuming the presence of three lifetime components. The shortest one was ascribed to the mixture of annihilation of para-positronium (p-Ps, the singlet state of Ps) and free positrons annihilated in the bulk of the tissue, the intermediate one—associated with annihilation of free positron and the longest-living one, ascribed to o-Ps annihilation. The lifetime τ_1_ is longer than expected 0.125 ns for p-Ps, and the intensity I_1_ is higher than predicted by the theory—the intensities ratio I_1_:I_3_ = 1:3 is not maintained. A similar situation is known, among others for water^39^—the main component of tissue samples. Annihilation of some positrons in Kapton film was taken into account. With optimized measurement time (1.5 h), in the adopted analysis, Fit Variance is better than 0.96. Spectrum analysis was also carried out assuming the existence of two o-Ps components, as well as assuming the ratio I_1_: I_3_ = 1:3, and assuming the set value of τ_1_ = 0.125 ns. None of the approaches gave better Fit Variance.

### Histopathology (HS)

Tissue samples obtained at surgery were fixed in 10% buffered formalin and processed routinely through dehydration with graded alcohol and acetone, clearing with xylene and embedding in paraffin blocks. Next, 3 µm-thick sections were cut and stained with H&E. Each case was diagnosed according to the WHO Classification of Tumours of Female Reproductive Organs 2014 (High grade serous carcinoma, ICD-O code 8461/3; Leiomyoma, ICD-O code 8890/0) using a microscope Olympus BX46 and photographic documentation was prepared with a built-in digital camera. Each sub-sample, after antimatter probe examination, was also fixed in 10% buffered formalin, embedded in a paraffin block, cut into 3 µm-thick sections and stained with H&E.

### Correlation of PALS and focused histopathology (FHS)

**Figure 2:**
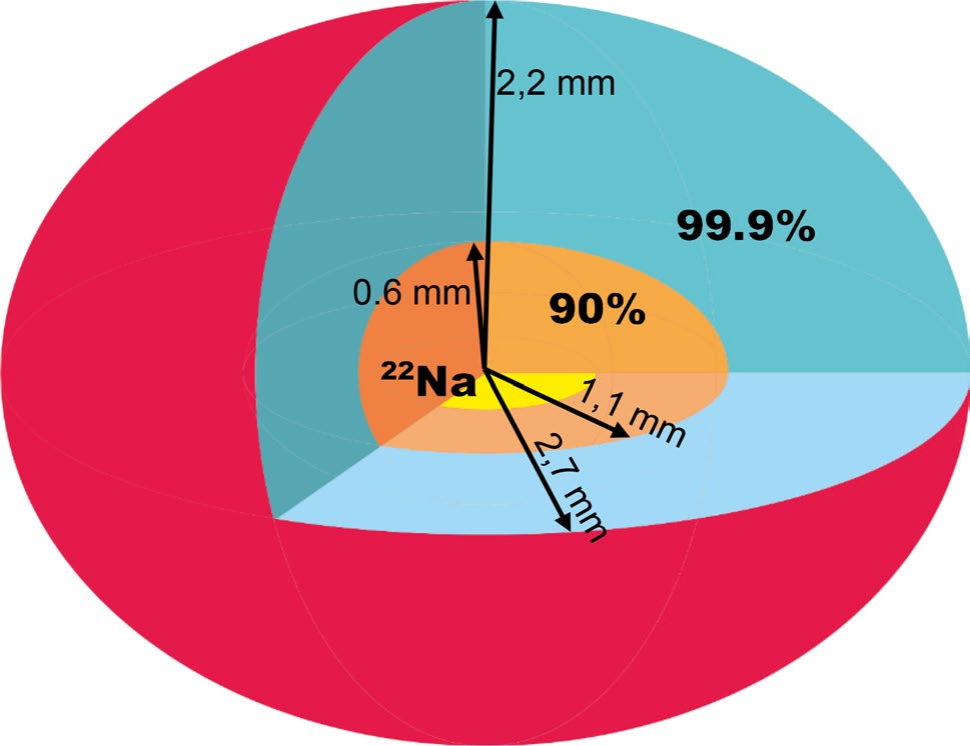
Schematic presentation of the e+ range in the sample. In the central part there is ^22^Na salt (flat dot, yellow), from which e+ are emitted in all directions. The positron's penetration area is marked: 90% of positrons is stopped at maximal distance of 1.1 mm from the source (orange area), and 99.9%—at 2,7 mm (blue area).

**Figure 3:**
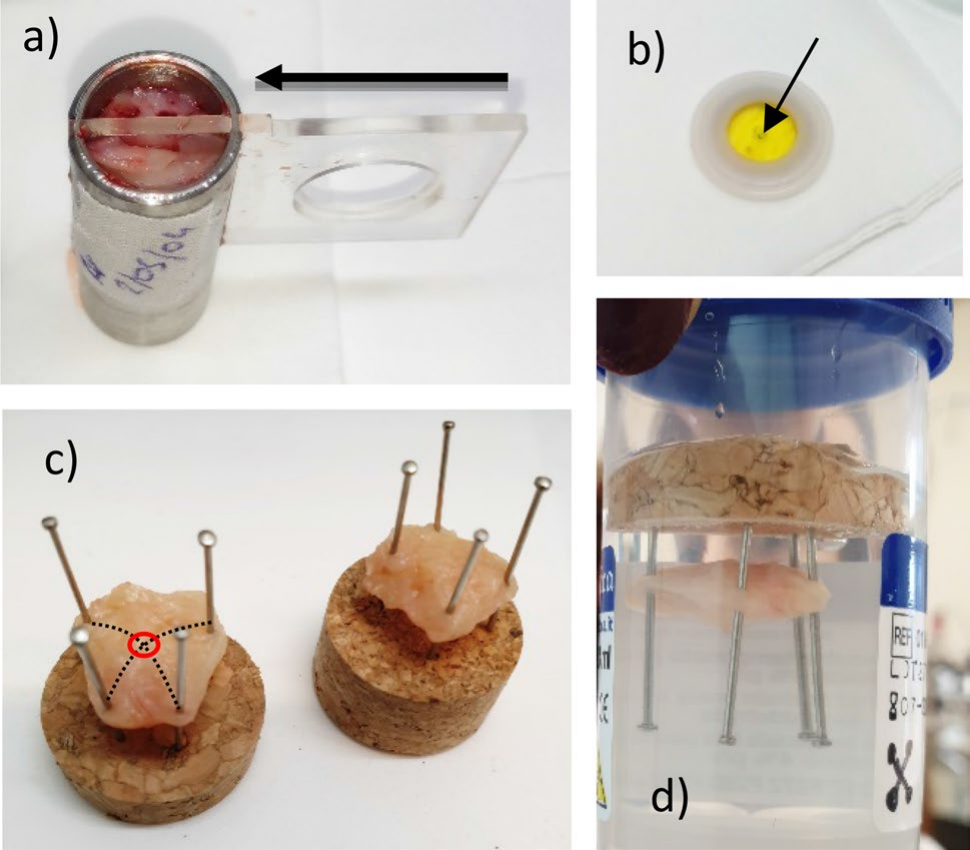
Handling of the samples: (**a**) measuring chamber with the two sub-sample placed there by the surgeon separated by a polycarbonate plate with a visible hole for placing the source, the direction of plate movement is indicated by an arrow, (**b**) the ^22^Na source in the Kapton foil and Teflon frame, (**c**) biological sample immobilized on a cork base, (**d**) sample placed in formalin.

Pilot studies performed for more than 40 samples have shown that standard histopathological analysis (HS) does not allow accurate correlation with PALS results in each case. The area of tissue viewed during histopathology is too large compared to the area examined by the antimatter probe. In matter of density 1 g/cm^3^, 90% of positrons do not penetrate deeper than 0.6 mm and 99.9% to 1.7 mm^1^. The technology used to prepare the ^22^Na sources allows to obtain a flat dot of activity not exceeding 1 mm in diameter spread over the surface of the Kapton film. Two pieces of the sample (named here sub-samples) surrounding the source constitute one sample. The range of positrons is presented schematically in Fig. 2: 90% of positrons penetrate 5 mm^3^ of the sample, the 99.9%—82 mm^3^. In the chamber, about 1.5 ÷ 2 cm^3^ of the sample (Fig. 3a) was placed. The ^22^Na is marked with a small dot in the center of the yellow Kapton film of positron source (Fig. 3b). As standard in histopathology, 2 × 2 cm slides are viewed. This proved to be the main reason for the discrepancy of first histopathological findings with both the PALS results and the surgeon's macroscopic qualification. Therefore, developing a methodology for the correlation of PALS studies with histopathological imaging has become a significant problem that has to be solved.

**Figure 4:**
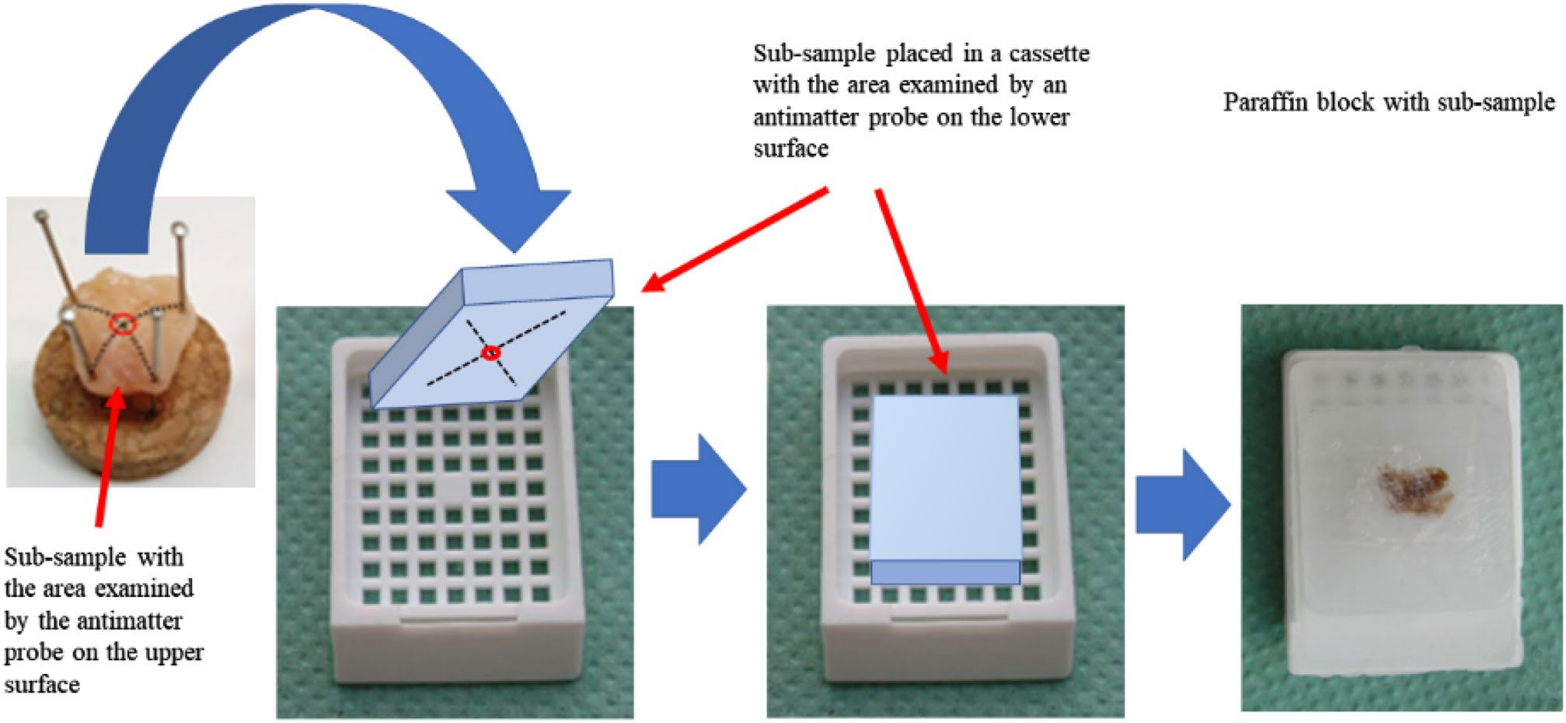
Scheme of embedding the sub-sample into a paraffin block.

Samples examined by the PALS technique for further histopathological analyzes began to be examined with an unambiguous location of the place that was penetrated by positrons—the point of the sample located at the intersection of the pins immobilizing the sample on the cork base clearly determined the area penetrated by positrons (Fig. 3c). Thus immobilized sample was placed in formalin and forwarded for further histopathology analysis (Fig. 3d). The middle point of the section was marked with a black marker and examined under a microscope. This point corresponded to the area examined earlier by the antimatter probe. The method of placing the sub-sample in the cassette is shown in Fig. 4. The focused histopathology imaging (FHS) more accurately describe the sample section penetrated by e+. Such precise recognition has been added to the standard SH description (see Table 1) and it is a new methodological approach to correlating the PALS—nanoscopic and histopathological—microscopic results.

**Table 1:** Selected information regarding the diagnosis and characteristics of the investigated samples.

Patient age	Clinical diagnosis	Sample number and sign of diagnosis	HS: standard histopathological diagnosis	FHS: focused histopathological diagnosis*
51	Uterine leiomyoma, right ovarian cyst	1H	S1** and S2: muscle: cross-section of the uterine corpus with the presence of smooth muscle tissue with cross-sections of thick-walled blood vessels	Smooth muscle tissue with cross-sections of thick-walled blood vessels
				Smooth muscle tissue with cross-sections of thick-walled blood vessels
		2H	S1: cervical muscle: cross-section of the cervical wall with the presence of smooth muscle tissue with cross-sections of small blood vessels and glands of the mucous membrane	Smooth muscle tissue with cross-sections of small blood vessels and cervical mucosa
			S2: cervical muscle: cross-section of the cervical wall with the presence of smooth muscle tissue with cross-sections of small blood vessels	Smooth muscle tissue with cross-sections of small blood vessels
		3A	S1 and S2: leiomyoma: cross-section of the leiomyoma	Leiomyoma with cross sections of blood vessels
				Leiomyoma with cross sections of blood vessels
		4H	S1: ovary: cross-section of the ovarian stroma	Ovarian stroma
			S2: ovary: ovarian cross-section with the presence of ovarian stroma and a fibrous connective tissue bands	Ovarian stroma with the fibrous connective tissue bands
66	Left ovarian tumor	5A	S1 and S2: left ovarian: high grade serous carcinoma of the left ovary	Tumor cells
				Connective tissue stroma within the tumor
		6A	S1: left ovarian: fragment of fibrous connective tissue with the presence of high grade serous carcinoma	Tumor cells
			S2: left ovarian: fragment of fibrous connective tissue with a small amount of neoplastic tissue around the periphery	Fibrous connective tissue
		7H	S1 and S2: right ovarian: cross-section of the ovary with corpora albicantia	Corpus albicans
				Ovarian stroma
		8A	S1 and S2: the omentum: Adipose tissue and fibrous connective tissue with diffuse tumor foci, in addition, cross-sections of vessels and nerve fibers are present	Fibrous connective tissue and adipose tissue
				Adipose tissue

*Description of the central part of the section.

**S1, S2 means sub-sample.

### Ethical approval and informed consent

All procedures performed in this study were per the ethical standards of the institutional and/or national research committee and with the 1964 Helsinki declaration and its later amendments or comparable ethical standards. The Bioethics Committee at the Medical University of Lublin approved the experimental protocols and research, issuing the Consent of the Bioethics Commission No. KE-0254/329/2016. Informed consent confirmed by the patients' signature was obtained from all individual participants included in the study. The samples come from patients qualified for hysterectomy. Samples were obtained from patients during surgeries performed in Provincial Specialist Hospital of Cardinal Stefan Wyszyński in Lublin.

## Results and discussion

Among the most important conclusions from the earlier research^11–22^, it is worth pointing out that sample preparation and measurement conditions are of key importance for the PALS response (e.g. time, drying, storage in formalin—significantly modifies the results). In the case of measurements taken over time, effects related to sample irradiation and sample aging may occurs. Change of measurement conditions (e.g. vacuum, gas atmosphere, humidity)^19^ are also reflected in the PALS parameters. All that significantly affects the establishment of measurement conditions. If we eliminate (or correct) the influence of the above factors, we come to the most important conclusions that the results are always very individual (i.e. it is not possible to indicate the reference level of τ_o-Ps_ or I_o-Ps_ for healthy or sick samples for the whole population) and therefore, the measurements of diseased tissue should be always correlated with the results obtained for healthy tissues of the same patient.

Our earlier research^20,21^ allowed us to find a general tendency of PALS parameters changes that make possible to conclude about the occurrence of lesions in the sample. Investigation carried out for pairs of samples: leiomyoma + uterine muscle, taken just after surgery, without any treatment, measured under normal conditions and in a short period of time after the surgery, showed a general tendency in the correlation between the PALS parameters of the healthy (*H*) and the affected (*A*) sample:

1 $$  \begin{array}{*{20}l}    {\tau _{{1H}}  < \tau _{{1A}} } \quad \hfill & {I_{{1H}}  < I_{{1A}} } \quad \hfill  \\    {\tau _{{2H}}  < \tau _{{2A}} } \quad \hfill & {I_{{2H}}  > I_{{2A}} } \hfill  \\    {\tau _{{3H}}  < \tau _{{3A}} } \quad \hfill & {I_{{3H}}  > I_{{3A}} } \hfill  \\   \end{array}   $$

**Figure 5:**
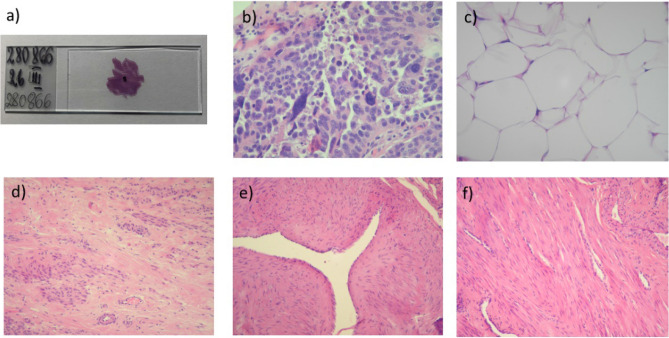
(**a**) Glass slide with section of sub-sample stained with H&E and midpoint of the slide marked with a black marker; (**b**) high grade serous carcinoma (H&E, 200 ×); (**c**) adipose tissue (H&E, 200 ×); (**d**) leiomyoma with fibrous connective tissue bands (H&E, 200 ×); (**e**) thick-walled arterial vessel (H&E, 200 ×); (**f**) leiomyoma with thin-walled venous vessel (H&E, 200 ×).

In this paper, the studies were carried out for 8 samples obtained during hysterectomy from two patients, the first one with diagnosed uterine leiomyomas and the second with the diagnosis: left ovarian cancer. Two sub-samples (S1, S2) were prepared for each sample according to the new focused histopathological standard (FHS). Samples, preserved in formalin after PALS measurements, were examined in accordance with the current standard and in accordance with the newly adopted methodology—descriptions of histopathology of slides of standard sizes (HS) and a section of the daughter penetrated by e+ (FHS) were prepared. The Table 1 summarizes the basic information about diagnosed patients along with an indication of additional information, including sample determinations. Photographs of selected samples obtained by the FHS method are presented in Fig. 5.

The histopathological (HS) result of both sub-samples (S1 and S2) for sample n^0^1, n^0^3, n^0^5, n^0^7, n^0^8 is the same. In the three remaining cases the sub-samples creating the sample for PALS tests differ from each other—their description in the Table 1 is divided into S1, S2. However, as one can easily see, histopathological examination of the tissue fragment penetrated by e+ (FHS) showed differences in 6 of 8 samples—only sub-samples in FHS imaging are the same, in sample n^0^1 and n^0^3. It means that positrons penetrate two different medium, and the obtained result will be a kind of averaging information about the nano-structure.

**Figure 6:**
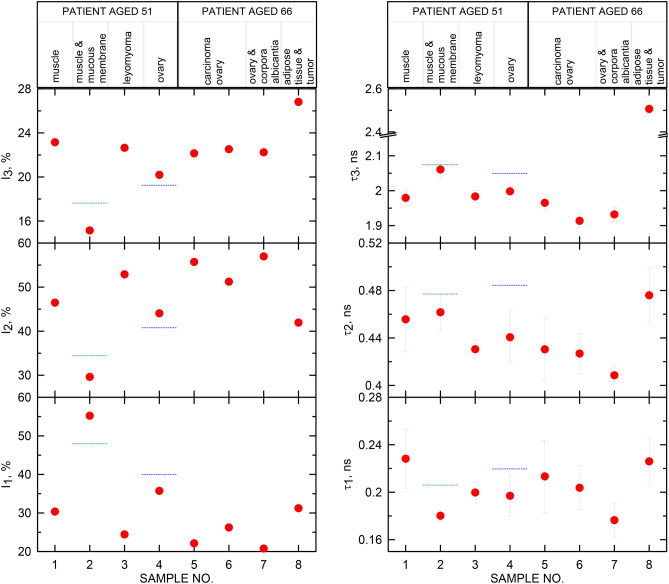
The intensities and lifetimes of mixed p-Ps and free e+ annihilation component (I_1_, τ_1_), free e+ component (I_2_, τ_2_) and o-Ps component (I_3_, τ_3_) in investigated samples. Red dots—results obtained using the PALS technique for the samples described in Table 1, dotted horizontal lines show examples of results obtained for samples from patients not described in this work, green line—the endometrium, blue line—ovarian sample.

The results of analyzes obtained by the PALS technique are shown in Fig. 6. The diverse nanostructure of healthy and diseased tissues should affect all PALS parameters, therefore Fig. 6 presents the changes of all measured parameters. The determined times τ_1_ and τ_2_ are burdened with relatively large errors, reaching up to 30 and 27 ps, respectively. The degree of complexity of the processes reflected by the two short-lived components is so large that a fourfold increase in spectrum statistics does not significantly improve the matching errors in the spectrum of these components.

Samples n^0^1 and n^0^2 are muscle tissue, but sample n^0^2 contains glands of the mucous membrane. Both tissues are healthy and come from the same patient, however, due to their origin in different parts of the uterus, they show a very large difference in nanostructure. The values of PALS parameters for cervical muscle (sample n^0^2) are clearly different from the results for the other samples. The o-Ps and free annihilation lifetimes are longer than in other investigated muscle tissues, while the shortest lifetime (τ_1_) is shortened to that of pure water^39^. At the same time, the intensity of this component (I_1_) is unexpectedly high, which reduces the intensity of the other components. Similar results were obtained by the PALS technique for other endometrial samples, as shown in Fig. 6. (green line, averaging the results obtained for atrophic endometrium sample and sample with growth endometrium). Samples of the uterine muscle and leiomyoma from the first patient (n^0^1 and n^0^3, respectively) cannot be unequivocally diagnosed by PALS due to the high concentration of blood vessels in all sub-samples (FHS is shown in Fig. 5d–f). The measured o-Ps lifetimes are the same in both samples, and only the proper correlation of intensities $I_{3H} > I_{3A}$ determined by Eq. (1) supports the identification of the diseased tissue. The incompatibility of short-lived parameters of the PALS spectra may have its roots probably in the increased content of iron atoms present in the positron penetration site (radical effects generated in the sample rich in blood vessels). The results obtained for a sample taken from the ovary (sample n^0^4) were first compared with the results obtained for another patient of similar age (52 years) and the diagnosis indicated uterine myomas and lesions in the ovary that manifested as abnormal vaginal bleeding (blue line in Fig. 6). The FHS analyzes have not been carried out for this reference patient. It is worth recalling here that the our first patient was diagnosed with numerous uterine myomas and left ovarian cysts. The FHS imaging showed that the positrons did not penetrate the cyst area, but ovarian stroma. It is difficult to determine whether the tissue penetrated by positrons is healthy or altered. Let us assume, however, that the Eq. (1) are true not only for fibroids, but also for other tissues, e.g. ovaries, and the patients of the same age and the same diagnosis can be compared. Then the PALS results obtained for these two patients will show that the reference patient's ovary shows lesions compared to the first patient's ovary. In Eq. (1) all relationships are true if we set PALS parameters for sample n^0^4 on the healthy tissue side.

Three samples taken from the second patient (n^0^5, n^0^6 and n^0^8) were diagnosed as cancerous. In three ovarian samples n^0^5, n^0^6 and n^0^7 some differences in PALS parameters are visible. Sample n^0^5 and n^0^6 come from an ovarian high grade serous carcinoma (FHS is shown in Fig. 5b), while sample n^0^7 is from an ovary considered healthy. The HS diagnosis showed that samples n^0^5 and n^0^6 are identical and tumor cells are present, however, the FHS diagnosis showed that one of the sub-samples of sample n^0^6 contains connective tissue in the e+ penetration area, thus positrons penetrated one cancer sub-sample and a second sub-sample with healthy tissues. The FHS result well justifies the PALS result and explains the differences in the PALS parameters in these samples. In the sample n^0^6 ("half-healthy"), all measured lifetimes are shorter than in the sample n^0^5. This means that the relation between the diseased and healthy sample (Eq. (1)), determined on the basis of number of altered uterus samples^20,21^, may also be true for another type of tissue—here the ovaries. The obtained result suggests a very high detection sensitivity of the PALS technique in relation to human tissue samples—even a slight difference in the concentration of identical lesions is reflected in the values of PALS parameters. Sample n^0^7 was diagnosed as a fragment of a healthy organ and PALS results confirm this preliminary diagnosis. Comparison of PALS parameters of the samples n^0^5 (A) and n^0^7 (H) shows compliance with Eq. (1). Figure 6 clearly shows that for sample n^0^8, the PALS parameters differ significantly from other ones. The HS and FHS analysis clearly indicate that e+ has penetrated the adipose tissue (FHS is shown in Fig. 5c). The density of adipose tissue^40^ is ~ 0.9 g/cm^3^, while the muscle tissue^41^ has a density of 1.06 g/cm^3^. The density of this sample lower by as much as 18% in comparison with muscle tissue can explain the clearly longer lifetime of all components and the much higher o-Ps intensity.

## Conclusions

Comparing the results obtained in the nano-scale using an antimatter probes and on a micro-scale histopathological diagnosis methods (HS and FHS) proved the Positron Annihilation Lifetime Spectroscopy to be a suitable technique for cancerous tissues investigations. However, this can be achieved only under the condition of the exact determination of the place of annihilation. It was additionally shown, that in contrast to histopathological methods, the ortho-positronium is a very sensitive probe that allows for determination of spatial distribution of abnormal cells inside human tissues. The relationship of PALS parameters determined between H and A tissues based on the results obtained for muscle tissues and uterine myoma described by Eq. (1) may be true not only for these tissues. That makes combining PALS with whole-body PET techniques, having high spatial resolution, very promising.

Showing the correlation of changes occurring in the sub-nanometric structure with the response of the Ps probe will affect the perception of mechanisms leading to the formation of neoplastic changes and will significantly contribute to improving the state of knowledge about the interaction of e+ and Ps with human tissues. However, the inhomogeneity of tissues obtained from a patient during surgery excludes this kind of samples as suitable for measurements leading to exact explanation of carcinogenesis issues, because of overlapping of many complex processes. Therefore, further research steps should proceed on two paths: (1) further systematic examination of various tissues and (2) research on various types of abnormal cells cultured on genetic cell lines. The second path will allow to lower the degree of cell diversity and contribute to a better understanding of carcinogenic processes.
